# Malignant Catatonia Mimics Tetanus

**DOI:** 10.5811/cpcem.2018.7.38585

**Published:** 2018-08-15

**Authors:** Ichiro Hirayama, Ryota Inokuchi, Takahiro Hiruma, Kent Doi, Naoto Morimura

**Affiliations:** The University of Tokyo Hospital, Department of Acute Medicine, Hongo, Bunkyo-ku, Tokyo, Japan

## CASE PRESENTATION

A 70-year-old Japanese man with untreated depression but no history of trauma had fallen seven days prior to hospitalization. After the incident he developed disturbance of consciousness, and his speech gradually became incoherent due to masseter spasm. His vital signs on admission were as follows: blood pressure 97/53 mmHg; pulse 99 beats per minute; body temperature 37.8 °C; respiratory rate 15 breaths per minute; SpO_2_, 99% without oxygenation; Glasgow Coma Scale Eye opening 3, Verbal response 3, Motor response 2. Physical examination revealed a back abrasion, stupor, and spasmodic laughter (Image). Blood tests including markers of inflammation and creatinine kinase, urinalysis, cerebrospinal fluid, blood cultures, imaging, and electroencephalography findings were normal. Administration of human tetanus immunoglobulin, tetanus toxoid, and penicillin did not improve the patient’s symptoms. On day two, blood tests were normal; thus, we administrated 5 mg diazepam. After that, we observed remarkable improvement in the patient’s consciousness, trismus, and fever.

## DIAGNOSIS

Catatonia is found in 10% of psychiatric inpatients, but malignant catatonia (MC) is rare.^1^ Catatonia is mainly caused by primary psychiatric, neurologic, metabolic and drug-induced disorders, as well as brain injury.^2^ Catatonia is most commonly characterized by mutism, stupor, posturing, and hypokinesis.^3^ Fever and autonomic dysregulation due to MC often lead to fatal consequences,^4^ with a mortality rate exceeding 50%.^5^ Evidence suggests that MC represents a disturbance of dopaminergic and gamma-aminobutyric acid receptors,^6^ as administration of 1 – 2 mg lorazepam typically leads to rapid resolution of symptoms within two hours.^7^ Such treatment should be used within 24 hours after excluding alternative diagnoses.^8^ Because diagnosis is often difficult and delayed,^9^ administration of low-dose benzodiazepines (e.g., five mg diazepam) may be warranted in patients with a history of psychological disorders presenting with MC symptoms.

**CPC-EM Capsule**

What do we already know about this clinical entity?

Malignant catatonia (MC) often leads to fatal consequences. Administration of low-dose lorazepam typically leads to rapid resolution of symptoms; thus, definite diagnosis is crucial.

What is the major impact of the image?

Because MC resembles tetanus, diagnosis is often difficult and delayed.

How might this improve emergency medicine practice?

Administration of low-dose benzodiazepines may be warranted when patients presenting with MC symptoms have a history of psychological disorders and normal blood, urine, cerebrospinal fluid testing and imaging.

Documented patient informed consent and/or Institutional Review Board approval has been obtained and filed for publication of this case report.

## Figures and Tables

**Image:**
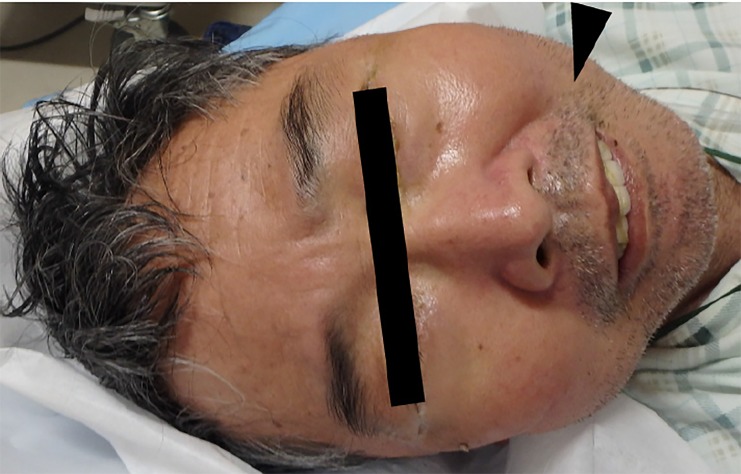
Patient shows sardonic smile (arrowhead) and stiff neck with fever and coma.
